# Deep Learned Quantization-Based Codec for 3D Airborne LiDAR Point Cloud Images

**DOI:** 10.3389/frobt.2021.606770

**Published:** 2021-05-13

**Authors:** A. Christoper Tamilmathi, P. L. Chithra

**Affiliations:** Department of Computer Science, University of Madras, Chennai, India

**Keywords:** nyquist signal sampling, min-max signal transformation, airborne spatial information, LiDAR, 3D point cloud image, deep learning, quantization, 3D image compression

## Abstract

This paper introduces a novel deep learned quantization-based coding for 3D Airborne LiDAR (Light detection and ranging) point cloud (pcd) image (DLQCPCD). The raw pcd signals are sampled and transformed by applying the Nyquist signal sampling and Min-max signal transformation techniques, respectively for improving the efficiency of the training process. Then, the transformed signals are feed into the deep learned quantization module for compressing the data. To the best of our knowledge, this proposed DLQCPCD is the first deep learning-based model for 3D airborne LiDAR pcd compression. The functions of Mean Squared Error and Stochastic Gradient Descent optimization function enhance the quality of the decompressed image by 67.01 percent on average, compared to other functions. The model’s efficiency has been validated with established well-known compression techniques such as the 7-Zip, WinRAR, and tensor tucker decomposition algorithm on the three inconsistent airborne datasets. The experimental results show that the proposed model compresses every pcd image into constant 16 Number of Neurons of data and decompresses the image with approximately 160 dB of PSNR value, 174.46 s execution time with 0.6 s execution speed per instruction, and proved that it outperforms the other existing algorithms regarding space and time.

## Introduction

A LiDAR is an active optical technique that creates the high-density 3D point cloud image of sampled Earth’s surface by transmitting the signal pulses toward the target image in the Earth, then, detects and analyses the signal from the target by receiver sensor in the LiDAR. The receiver sensor calculates the time interval between the signal pulse left from the sensor and the reflected signal received for finding the distance of the object to the ground (What Is Lidar Data Help ArcGIS Desktop, n.d). The LiDAR sensor record the information about the Earth as a pcd image and each point in the pcd holds some of the attributes like 3D spatial information (x, y, z), intensities, color values (red, green, blue), flight angle, etc. The resulting of the recorded point clouds are stored in the form of a laser file system (LAS) or point cloud system (pcd) format (What Is a Point Cloud What Is LiDAR, n.d). The LiDAR can generate 160,000 pulses per second; this will create a massive raw point cloud data. It is a very challenging task to store and analyze this huge data. A high efficient compression process is mandatory to solve this problem. In an earlier stage, the point cloud compression has been done by using octree and voxelization methods, then, it slowly moves to the tensor-based compression version. Now it reaches out to the artificial technology, to apply learning algorithms on the pcd image to compress the huge data. Nowadays many of the machine learning algorithms such as classification, detection, segmentation, and identification are focuses on the pcd images and are implemented on some of the airborne datasets. A very few countable point cloud autoencoders have been designed and tested only for balanced ModelNet and ShapeNet datasets but the machine learning-based compression model for airborne LiDAR pcd datasets is yet to be designed. Our proposed DLQCPCD network model is the first deep learned-based codec model for unbalanced airborne LiDAR pcd datasets.

This proposed point cloud compression work depends on the preprocessing methods and the deep learned quantization module. Some of the existing related works of DLQAEPCD are discussed in this section. The point cloud image surface has been represented by the characteristics of the sampled signal, generated by applying the Nyquist frequency rate value (Mineo and Summan, 2019). The mathematical manipulation formula helps to downsample the point cloud by multiplying the maximum signal value with a scaling factor, which is less than one, and round it to the closest integer coordinate value (Wang et al., 2019). In the traditional octree downsampling algorithm, convert the leaf node values as the patches of pcd image for further processing steps (Golla and Klein 2015). In the high-density point cloud image, the unnecessary points are removed and retaining the sparse points in the planar, cylindrical, and rough neighborhood areas (Lin et al., 2016). The down-sampled pcd points are transformed into the range of some values for reducing the complexity of the data manipulation. One of the probability-based, improved normal distributive transformation methods has been applied to the point cloud image to normalize the points (Merten 2008). The statistical-based, multi-directional affine registration algorithm transforms the pcd data values to suitable data for the registration process (C. Wang et al., 2018). On the other way, the geometric information of pcd data has been transformed based on quadratic constraints, which have combined the point’s orientation and position of the line features (Sheng et al., 2018).

In the earlier compression techniques, the maximum of pcd preprocess methods only based on the voxel grid or octree algorithm. Some of the preprocess work based on the segmentation, which is based on the region growing segmentation process in which the discarded boundary points are restored by using polynomial equations of degree one during the decompression process (Imdad et al., 2007). The combination of plane fitting and discrete wavelet transform algorithm improves the quality of the reconstructed pcd image (Chithra and Christoper 2018). On the other hand, Tensor based point cloud compression algorithm has been efficiently reduced the storage space, then perfectly reconstruct the original point cloud image (Chithra and Tamilmathi, 2020b). The dimension of 3D point cloud data has been reduced into a single order tensor to minimize the storage space and transmission time by applying the Tucker decomposition method (Chithra and Tamilmathi, 2020a). Recently Artificial Intelligence is made a greater change in the point cloud process. Machine learning networks face real-time challenging tasks is to handle the point set that directly taken from the point cloud image. 3D point cloud has been classified by using high-performance transfer learning algorithms (Zhao et al., 2020). A 3D point cloud image has been classified and segmented by using a structure-aware convolution neural network (Wang et al., 2020). The geometric information of point cloud data has been preserved by applying the spectral decomposition filter and produces a good performance in the point cloud registration process (Labussiere et al., 2018). Nowadays, some machine learning-based algorithms focus on the 3D autoencoder development process for encoding and reduce the dimension of the point cloud image. Only a few countable machine learning algorithm based model concentrates on the 3D point cloud compression work. Pointnet-based deep autoencoder algorithm replaced the transformation function in the point cloud compression technique (Yan et al., 2019). The structure of the 3D point cloud image has been compressed by using the 3D convolutional layer model (Quach et al., 2019). Voxelized and scaled, non-overlapped 3D cube structure point cloud fed into the stacked convolutional network to improve the latent feature characteristics of the pcd image (Bello et al., 2020). Another type of sparse autoencoder and compressed sensing method improves the speed of the reconstruction process (Chen et al., 2019). The quality of the reconstruction point cloud image has been improved by the folded neural network with a tuned weight model (Wang et al., 2012). The effective latent code has been created from the convolution network model by maintaining the adaptive features of the image (Yuhui et al., 2019). The quality of the actual pcd image is compared with the target image by different quality metrics (Schwarz et al., 2019).

This proposed DLQCPCD work compresses the spatial information of airborne LiDAR pcd image based on the deep learned quantization algorithm. First, the unbalanced raw pcd data are sampled by applying the Nyquist signal sampling technique. Then, the sampled signal data are transformed by using the Min-max signal transformation method. The deep learned quantization model has taken the transformed signal data as the input and produces the latent vector as a compressed form of bitstream data. This model has been implemented and tested on three different dense airborne LiDAR pcd datasets and compared with the existing algorithms.

Our main contributions are described in two folds.

1. Quantization is a core function of the traditional compression procedure. The quantization and dequantization modules have been replaced by the deep learned quantization network structure to increase the compression ratio and the quality of the reconstructed image with high speed.
2. The above-discussed autoencoder and deep learning-based compression models have been created only for a balanced Terrestrial synthesis ModelNet and ShapeNet datasets in OFF format files. These two data sets are different from our unbalanced, unlabeled airborne LiDAR data set. So far there is no model is available for our proposed 3D airborne LiDAR pcd datasets. Other machine learning algorithms such as segmentation, detection, identification, and classification methods are implemented in the airborne dataset. To the best of our knowledge, this is the first proposed compression model for 3D airborne LiDAR pcd datasets based on a deep learning algorithm.

Experimental results show that the proposed DLQCPCD algorithm compresses every pcd image into constant 16-bits of data and the quality of the reconstructed image averagely increased by 67.01% on average compared to the other function combination. This is the first deep learning-based model implemented on 3D airborne LiDAR pcd image compression. The compression performance and the compression efficiency of the proposed DLQCPCD model are compared with the existing well-known compression algorithms such as 7-Zip, WinRAR, and tensor tucker decomposition algorithm, respectively. The experimental results show that the proposed model compresses every pcd image into 16 Number of Neurons of data and decompresses the image with approximately 160 dB of PSNR value, 174.46 s execution time with 0.6 execution speed per instruction and proved that it outperforms the other existing algorithms regarding space and time complexity.

This paper is organized as follows. *Proposed Deep Learning-based Compression Methodology* presents the proposed deep learning-based compression methodology. The datasets and the experimental results are discussed in *Experimental Results*. Finally, the conclusion of the work is given in *Conclusion*.

## Proposed Deep Learning-based Compression Methodology

**FIGURE 1:**
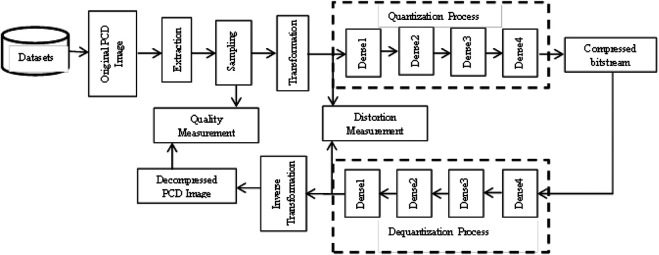
The architecture of the proposed DLQCPCD algorithm.

The architecture of the proposed DLQCPCD method is shown in Figure 1. The proposed compression process consists of three steps; i) Nyquist Signal sampling, ii) Min-max Signal transformation, and iii) Deep learning-based quantization process. A detailed explanation of the proposed DLQCPCD is given below.

### Nyquist Signal Sampling

The massive, continuous pcd signal has been discretized into finite signal data by using the Nyquist sampling technique. This sampling technique supports the distortion-free reconstruction process. The main aim of this Nyquist sampling method is to select the discrete sequence of signal values to get the complete information from the continuous signal by using the Nyquist sample rate. This sampling method does not lose any information in the original point cloud. Millions of signal pulses have been recorded by the LiDAR sensor per second hence the raw data is very huge and also each pcd image in the dataset has a varying size of 3D point data. It is not efficient to train the single model for all pcd images in the dataset. Thus the imbalanced data in the dataset should be balanced by the Nyquist sampling function before feed into the training process. The three different coordinate signal pulses x, y, z have been independently sampled by the constant sample period *δ*_*s*_. Then, the Nyquist sampling frequency (Nyquist sampling rate) *χ*_*s*_ can be represented as the following Eq. 1.

$$X_{s}=\frac{1}{\delta_{\text{s}}},$$ (1)

The Nyquist sampling rate denotes the number of samples is taken for the further process. The Nyquist sampling theorem denotes that the frequency is strictly less than half of the sample rate. In this proposed method the constant sampling period *δ*_*s*_ *=2*. The discrete signal samples have been collected by using this sample period from the Nyquist rate (2*δ*_*s*_) of the continuous signal. Hence the alternative signal samples are collected from the original recorded signal. Then the Nyquist sampling rate is half of the portion of the original signal that has been taken as a sampled signal data of the original signal. In the pcd signal, *S*_*p*_ expressed in Eq. 2.

$$S_{p}=\left\{X_{n},Y_{n},Z_{n}\right\},$$ (2)

$$X_{\text{n}}=\left\{x_{1}{,x}_{2}{,x}_{3}\text{,}...{,x}_{\text{n}}\text{,}\right\},$$

$$Y_{\text{n}}=\left\{y_{1}{,y}_{2}{,y}_{3}\text{,}...{,y}_{\text{n}}\right\},$$

$$Z_{\text{n}}=\left\{z_{1}{,z}_{2}{,z}_{3}\text{,}...z_{\text{n}}\right\},$$

where *X*_*n,*_ *Y*_*n,*_ *Z*_*n*_ are the set of x, y, z coordinate values, respectively. The *n* is a number of signal data in each set. From each set, i positioned data have been collected for sampling, and the remaining (i−1) positioned signal data are not considered for further processing. Hence the *n/2* sampling rate signal data only consider for the next transformation process.

### Min-Max Signal Transformation

The real-world coordinate values of recorded LiDAR pcd data have been transformed into the window (standard) coordinate values to improve the training stability of the described model. This standard transformation has been done by the Min-max pulse transformation function. This proposed Min-max transformation is one of the efficient and fewer computation methods to transforms the raw coordinate pulse value into the range of 0 and 1 without affecting the structure of the pcd image. In this transformation, the minimum pulse value of the signal is transformed into 0 and the maximum pulse value of the signal is transformed into 1. The other pulse value of a signal is transformed in between the range of 0–1. The main goal of this transformation algorithm is to move every signal to the same scale to make them equally involved in the further processing technique. The transformation function is described by the following equations

$$\omega_{\max}=MAX\left\{S_{p}\right\},$$

$$\omega_{min}=MIN\left\{S_{p}\right\},$$

$${S^{\prime }}_{p}=V_{i=1}^{n}\left(x_{i}-\omega_{min}\right)/\left(\omega_{max}-\;\omega_{min}\right),$$ (3)

where $x_{i}$, $\;\omega_{max}$ *,* $\omega_{min}$ are the i^th^ input signal, maximum and minimum signal value in the pcd signal set *S*_*p*,_ respectively that contains the different range of pulse value. *S*_*p*_ *′* is the transformed pcd signal values are normalized in the range of 0 and 1. The resultant normalized signal value improves the efficiency of the training process of the proposed model.

### Deep Learned Quantization

Quantization is the process of mapping the massive raw pcd data into a minimum number of the necessary bitstream for the storage and transmission process. DLQ is a deep learning-based network to reduce the dimension of the normalized 3D structured pcd data into a single order tensor with a fewer number of bytes to reduce the time and space complexity of the storage and the transmission process. The architecture of DLQ is shown in Figure 1, which consists of a quantization module with encoder function *c = Q*_*θ*_ *(x)* and a dequantization module with decoder function *x’ = D*_*φ*_ *(c)* where *x, x’, c, Q,* and *D* are input variable, decoded variable, latent space vector (compressed bitstream), quantization function and dequantization function, respectively. The DLQ network has been trained by the optimized encoding and decoding parameters *θ* and *φ*. Every 3D point in the pcd image has been quantized by using multiple dense layers in the DLQ network. The quantization module has been constructed by four dense layers with 128, 64, 32, 16 neurons, respectively followed by ReLU activation function. ReLU activation function selects the necessary information from the image for the compression process. It produces better performance in the proposed model compared with the other optimized activation functions like linear and sigmoid activation functions. The deep learning-based quantization function is denoted by Eq. 4.

$$Q=D_{4}\left(D_{3}\left(D_{2}\left(D_{1}\left({S^{\prime }}_{p},Q_{e1}\right)Q_{e2}\right)Q_{e3}\right)Q_{e4}\right),$$ (4)

where *S*_*p*_ *′* is a normalized input pcd set with n number of 3D points in the spatial domain, which is an input and target output data of deep learning module. *Q* is a quantized bitstream (Latent space vector) with a constant compressed size of pcd image, which is a resultant stream of deep learned model with four fully connected layers *D*_*1*_ *, D*_*2*_ *, D*_*3*_, and *D*_*4*_ with the i^th^ optimized quantization parameters *Q*_*ei*_. The dequantization functional part is consists of four dense layers with 32, 64, 128, 6144 neurons followed by the ReLU function, and then the sigmoid function. The dequantization function has been restored the missing value in the latent space vector by applying the inverse process of a quantization process, which is denoted by the Eq. 5.

$$S_{p}^{\prime \prime }=D_{1}\left(D_{2}\left(D_{3}\left(D_{4}\left(Q,D_{e4}\right)D_{e3}\right)D_{e2}\right)D_{e1}\right),$$ (5)

where *D*_*ei*_ is an optimal dequantization parameter applied on a quantized bitstream to produce back to the normalized pcd data through the trained dequantization deep learning module. The output pcd signal of the dequantization module is denoted by *S*_*p*_ *′′.* In this deep learning architecture, each neuron in the layer linked with all the neurons in the successive layer through the link is called weight (*w*). The bias value is linked with all the neurons in each layer. This proposed quantization architecture has been deep learned by applying the Mean Squared Error (*MSE*) loss function to calculate the distortion between actual and targeted output pcd image is denoted in Eq. 6. This loss function leads to the Peak Signal-to-noize Ratio (*PSNR*) characteristics of the LiDAR pcd image.

$$MSE=\sum_{i=1}^{n}\frac{{\left(Sp^{\prime }\;-Sp^{\prime \prime }\right)}^{2}}{n},$$ (6)

Where *S*_*p*_ *′* And *S*_*p*_ *′′* are the target and actual output pcd image signal. The stochastic gradient descent (SGD) optimizer is applied to balance the weight values to reduce the distortion between the actual and target value of the model. Eq. 7 is describing the SGD calculation function.

Θ=Θ−(α×G), (7)

$$\Theta_{i}=\Theta_{i}-\alpha \left(S_{p}^{\prime }-S_{p}^{\prime \prime }\right)x_{j}^{i},$$ (8)

where *α* is a learning rate and *Θ*_*i*_ is an i^th^ random point selected for the gradient calculation, and G is a gradient value. The optimized hyperparameter values such as learning rate (α = 0.3) and momentum (β = 0.9) values are to increase the speed of the convergence rate. DLQ network has been trained by 6000 epochs to produce a better-reconstructed image but actually, the model reached convergence much earlier.

### Performance Metrics

The performance of the proposed DLQCPCD algorithm has been measured by objective quality metrics based on Point-to-Point (P2P) and Point-to-Plane (P2Pl) metrics. The distortion value between the actual output and the targeted output value has been measured by the chamfer distance (Yan et al., 2019). The sampled original pcd image is denoted by *V*_*org*_ and the decompressed pcd image is denoted by *V*_*deg*_. All the performance metrics are described in Table 1 (Schwarz et al., 2019)**.**

**TABLE 1:** The Objective quality metrics of 3D point cloud data.

Metrics	Formula
Root Mean Square Distance (RMSD) (P2P)	$d_{rms}\left(V_{org},V_{deg}\right)=\sqrt{\frac{1}{K}\sum_{vo\in Vorg}vo-vd\_nearest\_neighbour^{2}}$
Hausdorff distance (P2P)	$d_{haussdorf}\left(V_{org},V_{deg}\right)=\;max_{v_{o}\;\in V_{ogr,\;\;}}\left(\;||v_{o}-v_{d_{nearest_{neighbour}}}|{|}_{2}\;,\;v_{d}\;is\;the\;point\;in\;Vdeg\;closest\;to\;v_{o}\right)$
PSNR using RMSD (P2P)	$psnr\;drms=10\log_{10}\left(\left|255\right|{|}_{2}^{2}/{\left(d_{symmetric\;rms}\left(V\right)\right)}^{2}\right)$
PSNR using Hausdorff distance (P2P)	$psnr\;haussdorf=10\log_{10}\left(\left|255\right|{|}_{2}^{2}/{\left(d_{haussdorf}\left(V\right)\right)}^{2}\right)$
Chamfer Distance	$d_{ch}\left(P,\;P^{\prime }\right)=\max\;\left\{\begin{array}{c} \frac{1}{\left|P\right|}\sum_{x\;\varepsilon P,\;\;y\;\varepsilon \;P^{\prime }}\min||x-y_{2}||\\ \frac{1}{\left|P^{\prime }\right|}\sum_{x\;\varepsilon \;P^{\prime },\;\;y\;\varepsilon \;P}\min||x-y_{2}|| \end{array}\right\}$

## Experimental Results

**FIGURE 2:**
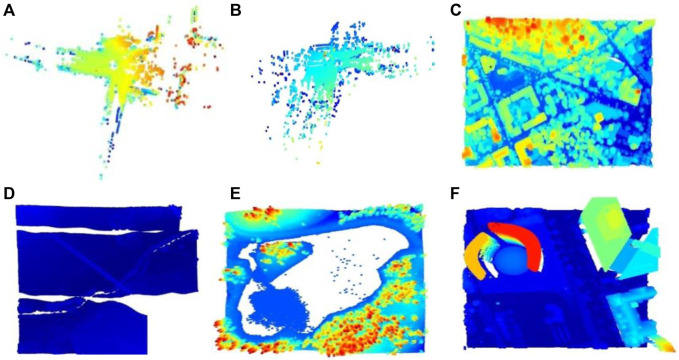
Input sample images from three different datasets **(A)** Scan2446(S) **(B)** Scan23124(S) **(C)** Csite3(I) **(D)** Fsite8(I) **(E)** Lake(L) **(F)** Building(L).

The proposed DLQCPCD implemented and tested on the three different size and dense, 3D Airborne LiDAR point cloud datasets using Jupyter environment in *Python* 3.7.3 on Windows 10 with 12.0 GB RAM and X64 bit processor. The first one is, the LAS format of a huge 3D LiDAR point cloud dataset (Downloads, n.d), which contains the seven different point cloud images (National Lidar Dataset - Wikipedia, n.d). The second one is, XYZ format of the Sydney Urban 3D object dataset (Sydney Urban Objects Dataset - ACFR - The University of Sydney, n.d), from that twenty-three massive scan data, have been trained and tested for DLQCPCD. The final one is, pcd format of the International Society of Photogrammetry and Remote Sensing (ISPRS) dataset, which contains the eight urban landscape-City Site (Csite) and rural landscape-Forest Site (Fsite) of the high-dense PCD data set (Test Sites, n.d). All the pcd in different datasets are converted into the unique pcd format. These datasets are split into the purpose of training (80%) process and testing (20%) process for efficiently evaluates the proposed model. Figure 2 shows some of the sample pcd images of three different LiDAR point cloud datasets.

**FIGURE 3:**
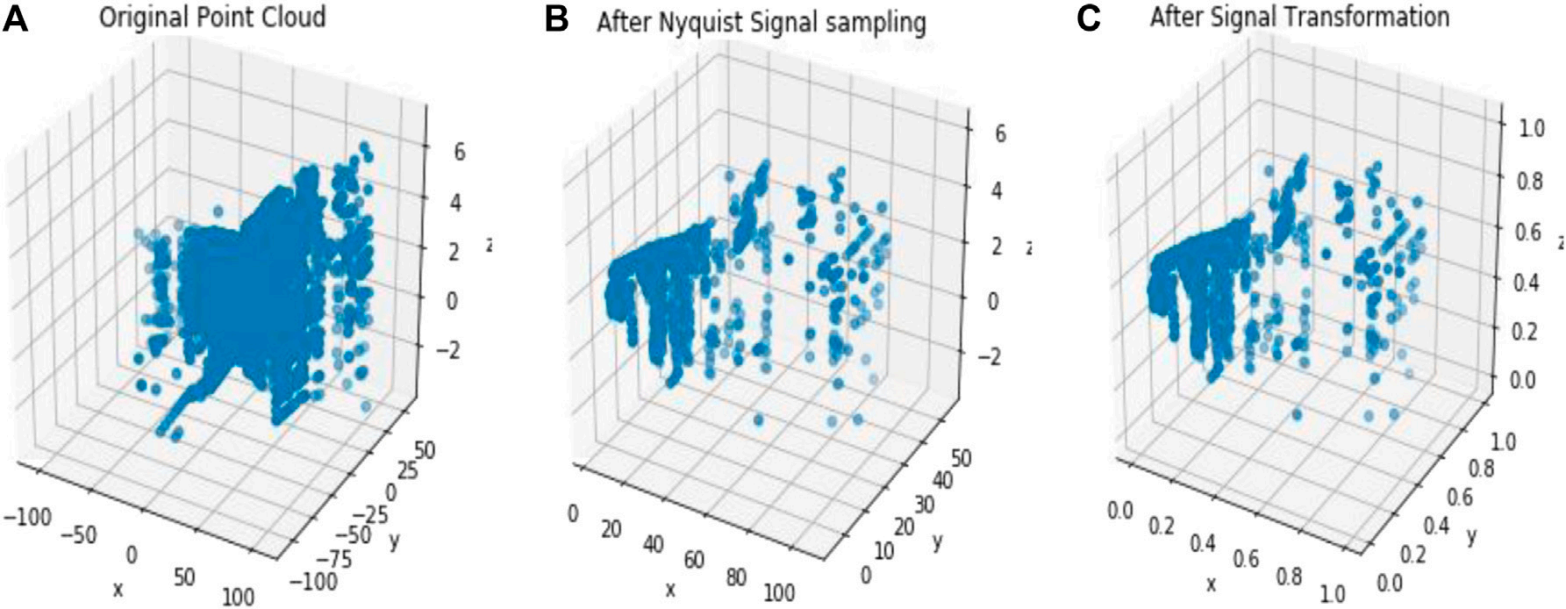
The output of the DLQCPCD preprocesses algorithm **(A)** Original Scan2446(S) pcd image **(B)** After Nyquist Signal Sampling **(C)** After Min-max Transformation.

In Figure 2, dataset names are defined by a single letter such as S for Sydney dataset, I for the ISPRS dataset, and L for the LiDAR dataset. The proposed compression method extracts only the spatial information from the different attributes of the pcd image for the compression purpose. This extracted inconsistent spatial information has been uniformly sampled by applying the Nyquist signal sampling technique on all the pcd in the datasets to increase the efficiency of the DLQ deep learning model. The sampling technique selects the 3D signal data based on the sampling rate (2048×3) and the sampling interval value (Two). One of the sampled Scan2446(S) point clouds is shown as a 3D scatter point graph in Figure 3B. Then the real-valued pcd image has been transformed into the window coordinate to reduce the computation complexity without affecting the structure of the point cloud. All the pcd data values are transformed into the range of 0 and 1 by applying the Min-max signal transformation method. This transformation technique is best suited for this DLCQPCD than the other transformation techniques. Figure 3C shows the 3D scatter point graph of the transformed point cloud. It illustrates that the range of the signal value is transformed without affecting the structure of the point cloud image.

**FIGURE 4:**
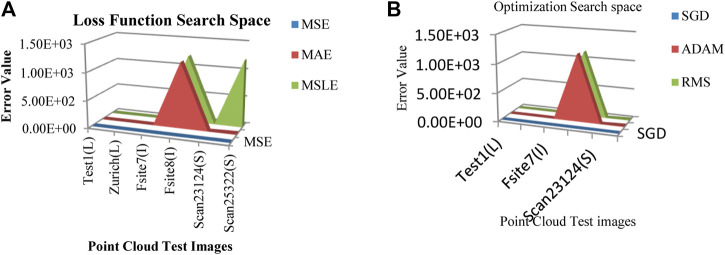
**(A)** Loss function Search Space **(B)** Optimizer function Search Space.

Next, the transformed values are fed into the input layer of the DLQ network which contains the quantization module with four different sized fully connected layers with 128, 64, 32, 16 neurons, respectively followed by the ReLU activation function. The last dense layer produces the latent vector as the compressed bitstream with 16 bits. The error values have been calculated and shown in Figure 4 for a different combination of functions. Figure 4A shows that the calculated error value while applying different loss functions like MSE, Mean Absolute Error (MAE), and Mean Squared Logarithmic Error (MSLE) on the datasets. From the graph, it is noted that the MSE loss function produces the minimum error value than the other functions. Hence, the MSE is selected as a suitable loss function for this proposed model.

Figure 4B defines that the calculated error value while applying different optimizer functions like SGD, ADAM, and Root Mean Square Properties (RMS) on the datasets. From the graph, it is observed that the function SGD produces a minimum error than the other functions. Hence, SGD is considered the best optimizer for the proposed model. The proposed DLQ network has been deeply trained by the MSE loss function and SGD optimizer to reduce the distortion between the actual and targeted output with less convergence time. The combination of MSE loss function and SGD optimizer function enhances the quality of the decompressed output image from the proposed model than the other combination of functions.

**FIGURE 5:**
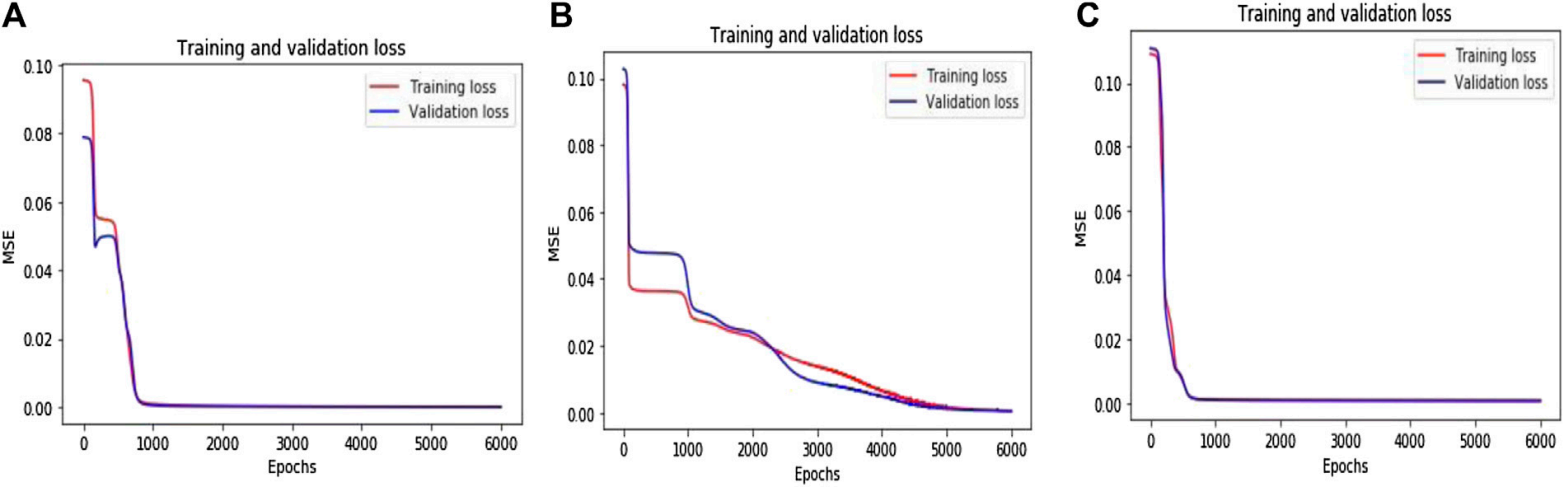
Training and validation loss value over the Epochs **(A)** 3D LiDAR pcd dataset **(B)** Sydney Urban 3D Object dataset **(C)** ISPRS dataset.

**FIGURE 6:**
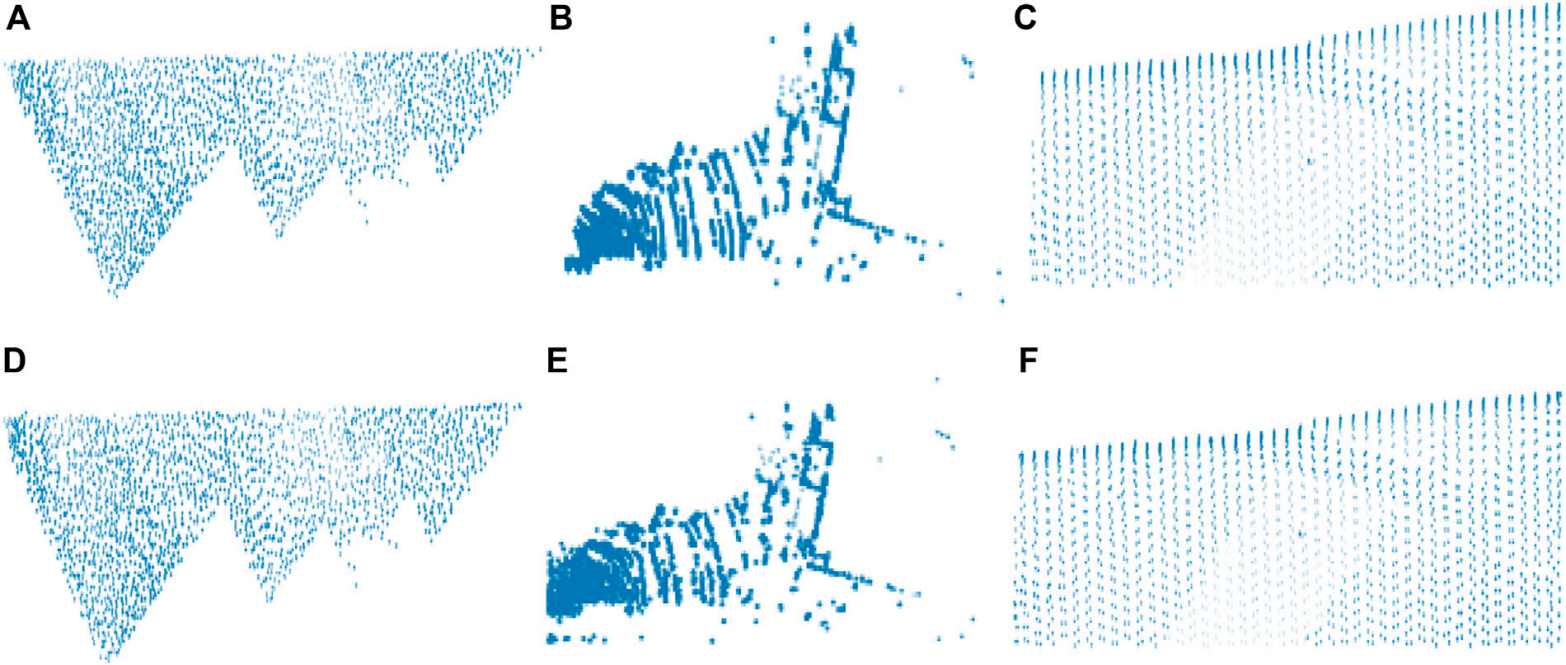
Target and the actual result pcd images of the DLQ model **(A)** Target Test1(L) **(B)** Target Scan19761(S) **(C)** Target Fsite8(I) **(D)** Actual Test1(L) **(E)** Actual Scan19761(S) **(F)** Actual Fsite8(I).

Figure 5 illustrates the training and validation loss values for three different pcd datasets. The proposed DLQ network trained by 6000 epochs for getting better quality reconstructed image but the model reached the convergence state much earlier that is shown in Figure 4. The DLQ network produces better target images that are shown in Figure 6. The sample target (input) and actual output pcd images from three datasets are shown in the Figures from 6**(A)** to 6**(C)** and from 6**(D)** to 6**(F)**, respectively.

The objective quality metrics in Table 1 are applied to an original and reconstructed image of the DLQCPCD algorithm; then the results are tabulated in Table 2. It shows only the quality metrics of two sample images from each dataset.

**TABLE 2:** The performance comparison between the different combinations of loss and optimizer functions on two sample images from each dataset.

Point Cloud Test Images	Optimization Function Search Space						Loss Function Search Space					
	PSNR			Hausdorff Distance			PSNR			Hausdorff Distance		
	SGD	ADAM	RMS	SGD	ADAM	RMS	MSE	MAE	MSLE	MSE	MAE	MSLE
Test1(L)	**139**	124.7	81.8	**0.02**	0.06	0.9	**139**	79.3	93.8	**0.02**	1.06	1.09
Zurich(L)	**128**	46.1	46.2	**0.07**	10.5	10.8	**128**	46.3	46.1	**0.07**	10.5	10.5
Fsite7(I)	**85.2**	93.3	53.1	**0.6**	2.08	9.19	**85.2**	64.8	55.6	**0.6**	6.1	24.9
Fsite8(I)	**121**	17.4	17.5	**0.1**	73.3	71.0	**121**	17.3	17.3	**0.1**	72.5	73.3
Scan23124(S)	**98.5**	115.5	62.2	**0.1**	0.1	0.6	**98.5**	63.2	71.3	**0.1**	1.8	5.8
Scan25322(S)	**122**	36.4	36.9	**0.1**	14.6	14.7	**122**	36.8	36.3	**0.1**	14.9	14.6

**FIGURE 7:**
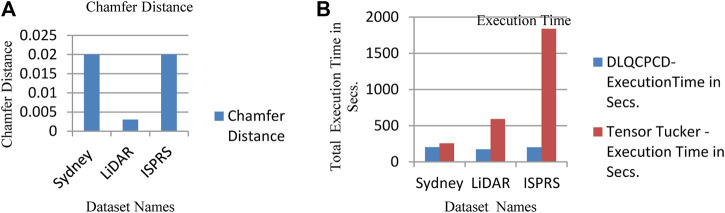
**(A)** The calculated Chamfer Distance of the Test Datasets **(B)** Comparison of Total execution time between proposed and Tensor Tucker compression algorithm.

From Table 2, it is proved that the MSE and SGD combination improves the PSNR value of the DLQCPCD algorithm’s decompressed image with minimum Hausdorff distance in all three datasets. The ADAM optimization function produces nearer to the value of the SGD function. In the loss function search space, the MAE function produces the very nearer value of the MSE function. The distortion-rate between the target and actual output of the DLQ model has been measured and shown in Figure 7A. In this Figure LiDAR dataset has less distortion rate than the other two datasets. Since, both the datasets are high dense than the LiDAR dataset.

The quality of the decompressed pcd from the proposed compression algorithm has been analyzed by using different objective quality metrics based on P2P and P2Pl methods. The different metrics formula has been mentioned in Table 1. The quality metrics Mean square error (MSE) and the Hausdorff mean square error (HMSE) for both P2P and P2Pl has been measured between the distance of original and decompressed image, is tabulated in Table 3.

**TABLE 3:** The objective distance measurement is based on Point-to-Point and Point-to-Plane metrics.

Point Cloud Name	Point-to-Point (P2P)		Point-to-Plane (P2Pl)	
	MSE	HMSE	MSE	HMSE
Scan11290(S)	1.49E-05	0.202613003	5.99E-11	0.000170152
Scan20631(S)	2.83E-09	0.138679112	6.73E-14	4.68E-05
Scan19761(S)	9.66E-08	0.189268328	2.99E-14	6.98E-05
Scan11886(S)	2.72E-07	0.119642347	8.20E-14	0.000137626
Scan2738(S)	4.58E-06	0.250359661	5.76E-12	9.96E-05
Fsite7(I)	0.0003987	0.632448537	3.22E-12	0.000320763
Fsite8(I)	0.0001926	0.361468333	3.62E-12	0.000259748
Test1(L)	1.29E-10	0.019763539	3.50E-16	7.29E-06
Zurich(L)	2.86E-10	0.078470708	5.13E-14	4.29E-05

**FIGURE 8:**
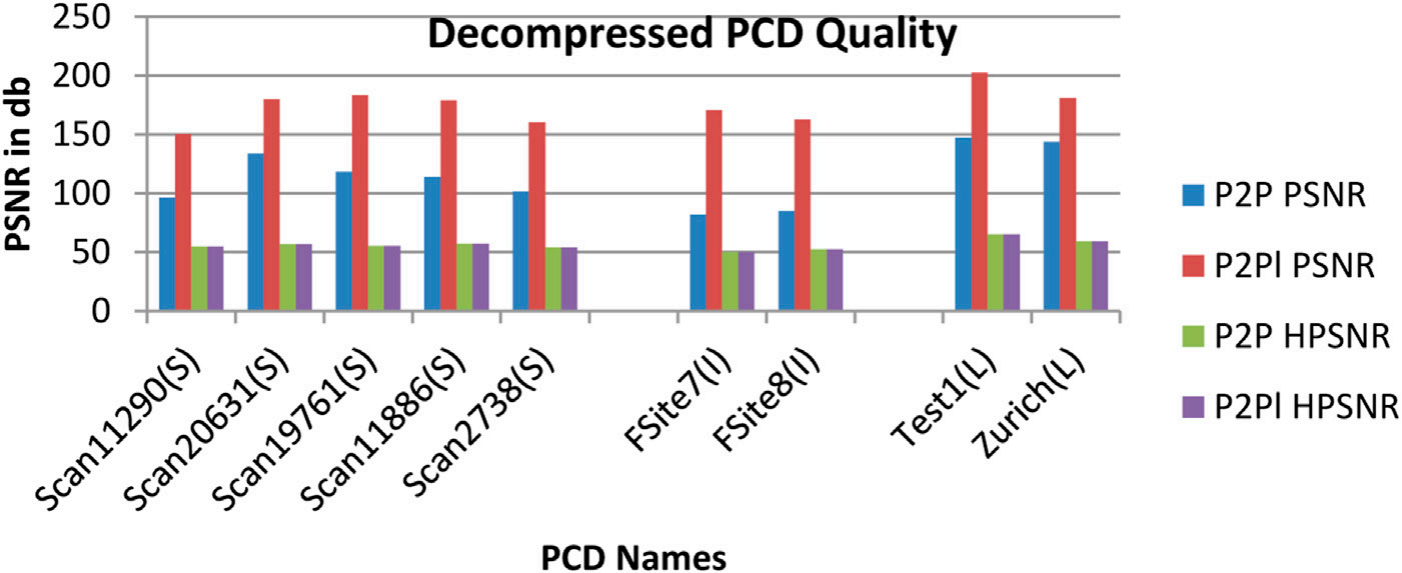
The PSNR value of the decompressed point clouds based on P2P and P2Pl.

From Table 3, it is observed that there is no noticeable distance between original and decompressed pcd. The quality of the decompressed pcd is measured by the Peak signal-to-noize ratio (PSNR) and Hausdorff peak signal-to-noize ratio (HPSNR). The calculated quality of the decompressed pcd from the proposed method is shown in Figure 8.

From Figure 8, it is observed that the Test1 point cloud from the LiDAR LAS dataset produced the high-quality decompressed point cloud rather than other point clouds. The performance of the proposed DLQCPCD algorithm is compared with the well-known general compression techniques (7-Zip and Win RAR) and the existing Tensor tucker decomposition algorithm (Chithra and Christoper 2018), (Chithra and Tamilmathi, 2020a), is shown in Table 4. The proposed well-trained deep learning-based architecture is to compress each point cloud from the three different databases into 16-bit compressed data. The existing algorithm can compress the single image at a time without having prior knowledge of the point cloud data. Hence, it takes more time to compress and decompress the point cloud. The proposed algorithm has gained knowledge during training the process. Once it is trained for the specific point cloud it can compress and decompress the set of point clouds within a minute.

**TABLE 4:** Comparison of compression performance between the proposed method and the existing algorithms.

Dataset Name	PCD Name	Location txt file size in KB	Existing Compression algorithm			Proposed DLQCPCD algorithm in Number of Neurons
			7-Zip in KB	Win RAR in KB	Tucker based on SVD in KB	
3D LiDAR LAS	Test1	507	78.9	81.7	29.3	16
	Zurich	28,224	429	459	31.3	16
Sydney Urban Objects	Scan 11290	1118	406	437	25.6	16
	Scan20631	1140	436	470	22.2	16
	Scan19761	1151	441	476	24.8	16
	Scan11886	1159	389	416	21.5	16
	Scan2738	1161	439	473	25	16
ISPRS	FSite7	6907	603	696	30.8	16
	FSite8	6082	423	490	31.2	16

From Table 4, it is concluded that the proposed deep learning-based model performed well with less distortion-rate at high speed than the existing Tensor Tucker compression algorithm. The existing well-known compression algorithm such as 7-Zip and WinRAR compresses the single pcd image into kilobytes (Chithra and Christoper 2018), (Chithra and Tamilmathi, 2020a), but this proposed DLQCPCD algorithm compresses every pcd image into 16 bits of the latent vector.

The proposed compression algorithm’s efficiency has been measured by some factors like Compressed point cloud, Quality of decompressed point cloud, Execution time, Execution speed, Main memory utilization, and Processor utilization. These factors are measured by testing the proposed algorithm and the existing tensor tucker decomposition algorithm with a 3D LiDAR dataset. The final calculated values are shown in Table 5.

**TABLE 5:** Efficiency comparison between the existing and proposed method.

Compression Efficiency Metrics	Existing Tensor tucker Algorithm	Proposed DLQCPCD Algorithm
Compressed bitstream	5.7% of the original image (averagely)	16 Number of Neurons
Quality of Decompressed PCD	55 db (average)	160 db (average)
Execution Time	591.8 s	174.46 s
Execution Speed (per instruction)	2.9 s	0.6 s
Main memory Utilization	3.8%	3.6%
Processor Utilization	44% (approximately)	60% (approximately)

Table 5 shows that the proposed DLQCPCD method achieves a high compression ratio, better quality of decompressed pcd, less execution time, less memory utilization with high speed than the existing tucker-based compression method with our system cofiguration.

Figures from 6 to 8 and Tables from 3 to 5, concluded that the proposed DLQCPCD lossy point cloud compression method produces better compression performance and compression efficiency than the existing algorithms. Hence, this efficient compress algorithm is suitable for LiDAR, Sydney, and Test site airborne datasets.

## Conclusion

In this work, a deep learned quantization-based codec has been developed for 3D airborne LiDAR pcd images. The Nyquist signal sampling and Min-max transformation algorithm have been applied on the raw pcd data to sampling and transforming the signal into the range of 0 and1 to increase the efficiency of the training process in the proposed algorithm. Then, the transformed data feed into the DLQ model to generate the latent code vector. The combination of MSE loss function and SGD optimization function improves the quality of the decompressed image by 67.01% on average compared to the other function combination. This is the first deep learning-based model implemented on 3D airborne LiDAR pcd image compression. The compression performance and the compression efficiency of the proposed DLQCPCD model are compared with the existing well-known compression algorithms such as 7-Zip, WinRAR, and tensor tucker decomposition algorithm respectively. The experimental results show that the proposed model compresses every pcd image into 16 Number of Neurons of data and decompresses the image with approximately 160 dB of PSNR value, 174.46 s execution time with 0.6 s execution speed per instruction and proved that it outperforms the other existing algorithms regarding space and time complexity. However, this proposed DLQCPCD compression work is developed only for spatial (geometry) information which is one of the seven attributes in the 3D LiDAR point cloud. The remaining attributes are occupied the same storage space as in the original point cloud. This algorithm can reduce only one attribute of the memory space in the original image based on the lossy compression technique.

## Data Availability

The original contributions presented in the study are included in the article/Supplementary Material, further inquiries can be directed to the corresponding author.

## References

[B1] Bello S. A. Yu S. Wang C. Adam J. M. Li J. (2020). Review: Deep Learning on 3D Point Clouds. Remote Sensing. 12 (11), 1729. 10.3390/rs12111729

[B2] Chen X. Zhou M. Zou L. Fan Li. Hu J. Geng G. (2019). A Fast Reconstruction Method of the Dense Point-Cloud Model for Cultural Heritage Artifacts Based on Compressed Sensing and Sparse Auto-Encoder. Opt. Quan. Elect. 51 (10), 1–16. 10.1007/s11082-019-2038-y

[B3] Chithra P. L. Christoper T. A. (2018). 3D Color Point Cloud Compression with Plane Fitting and Discrete Wavelet Transform. ICoAC. 2018, 20–26. 10.1109/ICoAC44903.2018.8939106

[B4] Chithra P. Tamilmathi A. C. (2020a). 3D LiDAR Point Cloud Image Codec Based on Tensor. Imaging Sci. J. 68 (1), 1–10. 10.1080/13682199.2020.1719747

[B5] Chithra P. L. Tamilmathi A. C. (2020b). Tensor Tucker Decomposition Based Geometry Compression on Three Dimensional LiDAR Point Cloud Image. IJITEE. 9 (3), 1897–1903. 10.35940/ijitee.c8551.019320

[B6] Downloads (n.d). “Downloads.” n.d. Available at: http://www.smartmm.com/downloads.html (Accessed September 5, 2020).

[B7] Golla T. Klein. R. (2015). “Real-Time Point Cloud Compression,” in IEEE International Conference on Intelligent Robots and Systems, Hamburg, Germany, September 28–October 2, 2015, 5087–5092. 10.1109/iros.2015.7354093

[B8] Imdad U. Asif M. Ahmad M. T. Sohaib O. Hanif M. K. Chaudary M. H. (2007). Three Dimensional Point Cloud Compression and Decompression Using Polynomials of Degree One. Symmetry 11 (2), 209. 10.3390/sym9030614

[B9] Labussiere M. Laconte J. Pomerleau F. (2018). Geometry Preserving Sampling Method Based on Spectral Decomposition for 3D Registration. Front. Robot., 1–15. Available at: http://arxiv.org/abs/1810.01666 (Accessed September, 2020). 10.3389/frobt.2020.572054 PMC7806074 33501332

[B10] Lin Y-J. Benziger R. R. Habib A. (2016). “Planar-Based Adaptive Down-Sampling of Point Clouds. Photogrammetric Eng. Remote Sensing 82, 955-966. 10.14358/pers.82.12.955

[B11] Merten H. (2008). The Three-Dimensional Normal-Distributions Transform. Available at: http://www.aass.oru.se/Research/Learning/publications/2009/Magnusson_2009-Doctoral_Thesis-3D_NDT.pdf (Accessed June 10, 2020).

[B12] Mineo C. Pierce S. G. Summan R. (2019). Novel Algorithms for 3D Surface Point Cloud Boundary Detection and Edge Reconstruction. JCDE. 6 (1), 81–91. 10.1016/j.jcde.2018.02.001

[B13] National Lidar Dataset - Wikipedia (n.d). National Lidar Dataset - Wikipedia. Available at: https://en.wikipedia.org/wiki/National_lidar_dataset (Accessed September 5, 2020).

[B14] Quach M. Valenzise G. Dufaux F. (2019). Learning Convolutional Transforms for Lossy Point Cloud Geometry Compression. ICIP. 2019, 4320–4324. 10.1109/icip.2019.8803413

[B15] Schwarz S. Preda M. Baroncini V. Budagavi M. Cesar P. Chou P. A. (2019). Emerging MPEG Standards for Point Cloud Compression. IEEE J. Emerg. Sel. Top. Circuits Syst. 9 (1), 133–148. 10.1109/jetcas.2018.2885981

[B16] Sheng Q. Wang Q. Hong R. Wang B. Zhang B. (2018). Geometric Transformation of Images and LiDAR Point Clouds under Quadratic Constraint. Remote Sensing Lett. 9 (10), 1011–1019. 10.1080/2150704x.2018.1499151

[B17] Sydney Urban Objects Dataset - ACFR - The University of Sydney (n.d). “Sydney Urban Objects Dataset - ACFR - the University of Sydney.” n.D. Available at: http://www.acfr.usyd.edu.au/papers/SydneyUrbanObjectsDataset.shtml (Accessed September 5, 2020).

[B18] Test Sites (n.d). “Test Sites.” n.D. Available at: https://www.itc.nl/isprs/wgIII-3/filtertest/downloadsites/ (Accessed September 5, 2020).

[B19] Wang C. Shu Q. Yang Y. Yuan F. (2018). Point Cloud Registration in Multidirectional Affine Transformation. IEEE Photon. J. 10 (6), 1–15. 10.1109/jphot.2018.2876689

[B20] Wang J. He H. Prokhorov D. V. (2012). A Folded Neural Network Autoencoder for Dimensionality Reduction. Proced. Comp. Sci. 13, 120–127. 10.1016/j.procs.2012.09.120

[B21] Wang J. Zhu H. Zhan M. Chen T. Liu H. Shen Q. (2019). Learned Point Cloud Geometry Compression. Comput. Vis. Pattern Recognit., 1–13. Available at: http://arxiv.org/abs/1909.12037 (Accessed September 26, 2019).

[B22] Wang L. Liu Y. Zhang S. Yan J. Tao P. (2020). Structure-Aware Convolution for 3D Point Cloud Classification and Segmentation. Remote Sensing. 12 (4), 634. 10.3390/rs12040634

[B23] What Is a Point Cloud What Is LiDAR (n.d). What Is a Point Cloud? what Is LiDAR?” n.D. Available at: https://community.safe.com/s/article/what-is-a-point-cloud-what-is-lidar (Accessed September 5, 2020).

[B24] What Is Lidar Data Help ArcGIS Desktop (n.d). What Is Lidar Data?—Help | ArcGIS Desktop.” n.D. Available at: https://desktop.arcgis.com/en/arcmap/10.3/manage-data/las-dataset/what-is-lidar-data-.htm (Accessed September 5, 2020).

[B25] Yan W. shao Y. Liu S. Thomas H. Zhu L. Ge L. (2019). Deep AutoEncoder-Based Lossy Geometry Compression for Point Clouds. Comput. Vis. Pattern Recognit., 4321–4328. Available at: http://arxiv.org/abs/1905.03691 (Accessed August 12, 2020).

[B26] Yuhui Z. Gutmann G. Akihiko K. (2019). Irregular Convolutional Auto-Encoder on Point Clouds, 1–20. Available at: http://arxiv.org/abs/1910.02686 (Accessed July 10, 2020).

[B27] Zhao C. Guo H. Lu J. Yu D. Li D. Chen X. (2020). ALS Point Cloud Classification with Small Training Data Set Based on Transfer Learning. IEEE Geosci. Remote Sensing Lett. 17 (8), 1406–1410. 10.1109/lgrs.2019.2947608

